# Primary appendiceal mucinous adenocarcinoma in two first-degree relatives: case report and review

**DOI:** 10.1186/1897-4287-9-1

**Published:** 2011-05-04

**Authors:** Adrianne R Racek, Kari G Rabe, Myra J Wick, Apostolos Psychogios, Noralane M Lindor

**Affiliations:** 1 University of North Dakota Medical School, 501 N. Columbia Road STOP 9037, Grand Forks, 58202-9037, ND, USA; 2 Division of Biomedical Statistics and Informatics, Mayo Clinic 200 First Street SW, Rochester, MN, 55905, USA; 3 Departments of Obstetrics-Gynecology & Medical Genetics, Mayo Clinic, 200 First Street SW, Rochester, 55905, MN, USA; 4 Kifisias Avenue, 3rd floor, Athens 115 23, Greece; 5 Department of Medical Genetics, Mayo Clinic, 200 First Street SW, Rochester, 55905, MN, USA

## Abstract

Carcinomas of the appendix are exceedingly rare tumors and have an annual age-adjusted incidence of around 0.4 cases per 100,000. Appendiceal adenocarcinoma accounts for < 0.5% of all gastrointestinal neoplasms and, of these, mucinous adenocarcinomas account for the majority. Published accounts of familial instances of primary appendiceal tumors are strikingly rare. We report two siblings who both developed primary mucinous adenocarcinomas. A genetics evaluation was conducted to determine if there was a recognizable underlying single gene disorder; no DNA mismatch repair defect was evident, and no other diagnosis was apparent. A review of appendiceal cancers seen at Mayo Clinic from l997 to the present was conducted to search for additional familial cases. Among 316 cases of primary appendiceal cancer of any histologic type, this sib pair was the only family reporting a second affected family member. The occurrence of appendiceal cancer in siblings may represent a random occurrence. An exceedingly rare predisposition syndrome cannot be ruled out.

## Background

Carcinomas of the appendix are rare tumors and have an annual age-adjusted incidence of 0.4 cases per 100,000 [1]. Appendiceal adenocarcinoma accounts for < 0.5% of all gastrointestinal neoplasms [2,3]. The diagnosis is rarely made preoperatively, leading one group of authors to claim no preoperative diagnoses have ever been reported [4]. While this may be an overstatement, it underscores the clinical experience that the vast majority of patients do not receive a preoperative diagnosis, but rather detection occurs either incidentally or due to an episode of acute appendicitis. An undiagnosed appendiceal cancer is reported to be found in about 1% of appendectomy specimens [5]. According to SEER data collected from 1973 to 2004, adenocarcinoma was the most frequently diagnosed appendiceal neoplasm reported in 65% of cases [2]. Due to the small sample size and range of clinical presentations, the diagnosis of appendiceal carcinoma remains challenging for physicians.

Very few reports have been published with familial appendiceal tumors. Anderson et al., presented a father-daughter case report in 1966. In this report, the ages at diagnoses were 42 and 15 respectively, and the histologies were reported as carcinoids of the appendix [6]. In 2001, Shih et al., presented a case of identical twins concordant for appendiceal mucinous adenomas. The symptomatic twin was 35 at age of diagnosis while the asymptomatic twin underwent elective appendectomy a few years later. Both histologies proved to be mucinous adenocarcinomas [7]. Because of the limited data, little is known about hereditary factors which may influence the development of appendiceal tumors. We present appendiceal adenocarcinomas occurring in siblings and review the Mayo Clinic experience with appendiceal cancers with an emphasis on searching for familial aggregation.

## Case Presentation

A 69-year-old white male of Greek descent presented to his local hospital with abdominal pain and nausea and was diagnosed with acute appendicitis. During appendectomy, the surgeon observed an appendiceal tumor. The pathology report confirmed a grade II mucinous adenocarcinoma involving the mucosa and extending up to the serosa. The patient developed a postoperative infection leading to his transfer to our tertiary care facility. Colonoscopy was conducted and no polyps or other tumors were found. A laparoscopic right hemicolectomy was performed and a follow up examination two years later showed no evidence of recurrent disease.

Two years later, the proband's sister, age 77 years old, presented with abdominal bloating and lower extremity edema. Pelvic ultrasound and CT scan revealed a pelvic mass, more than 23 centimeters in diameter. The CA-125 was elevated to 83.4 U/mL (reference range is < 35 U/mL) and the CEA level was 151.9 ng/mL (reference range < 3 ng/mL in adult nonsmokers such as this patient). Her brother, himself a physician, transferred her to our facility for gynecologic surgery consult. At the time of consultation, abdominal distention was so pronounced that she was unable to bend over and tie her shoes. Bimanual examination revealed a palpable pelvic mass which shifted uniformly across the abdominal cavity. A distinct adnexal mass was not palpable. Colonoscopy revealed only a 4 mm polyp in the transverse colon; pathology of the polyp indicated a tubular adenoma with low grade dysplasia. The patient elected to proceed to laparotomy. At laparotomy, the surgeons noted copious mucinous deposits as well as presence of a primary mucinous appendiceal adenocarcinoma that essentially replaced the entire right ovary. Due to the appendiceal tumor metastases, right salpingo-oophorectomy, ileoascending colon resection with side-to-side reanastomosis, and placement of intraperitoneal catheter were performed. A week postoperatively, a two-day course of 5-fluorouracil intraperitoneal chemotherapy was administered and completed.

Gross pathology was notable for a well-differentiated multiloculated mass 19 cm at the greatest diameter. Histologic evaluation revealed a well-differentiated mucinous adenocarcinoma arising in the background of a serrated adenoma with mucocele formation. The appendix contained a 6.0 X 2.5 X 2.0 cm mass that had invaded into submucosa, and involved the subserosal and periappendiceal soft tissue. The ileum and cecum contained no diagnostic abnormality while the sigmoid colon revealed acellular mucin. Immunostaining was performed on the paraffin-embedded tissue using antibodies directed against CK7, CK20, CDX-2, and estrogen receptor. The neoplastic cells showed strong cytoplasmic positive staining for CK20 with nuclear positive staining for CDX-2 and no significant staining for CK7 or estrogen receptor. These data support the diagnosis and indicate a colorectal (appendiceal) primary.

Because of the familial occurrence of these rare tumors in siblings, and because appendiceal cancer has been reported previously in Lynch Syndrome, this diagnosis was considered [8]. From the sister's tumor, no microsatellite instability was detected in five informative markers, and tumor immunohistochemistry showed normal expression of the four Lynch-associated gene products: MLH1, MSH2, MSH6, and PMS2. After his sister was diagnosed, the brother had a formal medical genetics evaluation for a possible hereditary cancer syndrome. His history was significant for bilateral renal cysts as well as an elevated baseline creatinine level of 1.6 mg/dL (reference range 0.8-1.3 mg/dL). A detailed family history was gathered and notable for distant consanguinity (parents were third cousins), maternal history of hemorrhagic stroke with questionable intracranial aneurysm, maternal uncle with bladder cancer, an aunt with pancreatic cancer, and maternal grandfather with prostate cancer. His sister and maternal uncle were both found to have bilateral renal cysts.

**Figure 1:**
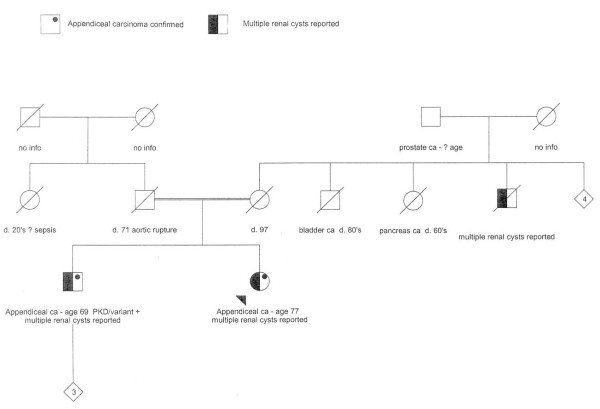
**Pedigree of sibling with appendiceal carcinoma**. Parents were third cousins.

Genetic testing was performed for autosomal dominant polycystic kidney disease (*PKD1, PDK2*). A novel variant of unknown significance was found in *PKD1*, an A > G transition at nucleotide position 2427 (codon position 739) which causes an amino acid change from glutamine to arginine in *PKD1*. DNA mismatch repair genes (*MLH1, MSH2, MSH6*) were sequenced and large rearrangement testing was conducted by multiple ligation probe assay (MLPA). The DNA repair genes were all normal (Figure 1 shows pedigree of this family).

In light of this sib pair concordant for appendiceal carcinomas and the paucity of literature on the subject, we conducted a retrospective chart review using the medical records at Mayo Clinic Rochester. Between l997 and July 2010, we identified 316 individuals with documented cancers of the appendix (314 plus the sib pair reported). We recorded the histologic type of cancer, the age at diagnosis, and looked for a family history of appendiceal cancer. The results of this chart review are shown in Table 1. For comparison, we also extracted data on ages at diagnosis and histology of appendiceal cancers from the SEER registry, which samples the population of the United States in multiple geographic locations (Surveillance Research Program, National Cancer Institute, http://www.seer.cancer.gov/seerstat version 6.6.1.). The summary of that data extraction is shown in Table 2.

**Table 1:** Characteristics of 316 appendiceal cancer cases seen at Mayo Clinic between 1995 and July 2010

	ALL CASES n = 316	MALES n = 131	FEMALES n = 185	P-VALUE
Positive family history, n (%)	0 (0)	0 (0)	0 (0)	1.0

Histology, n (%)				**0.01**
- Neuroendocrine/Carcinoid	120 (38)	42 (32)	78 (42)	
- Adenocarcinoma	185 (59)	88 (67)	97 (52)	
- Other	11 (3)	1 (1)	10 (5)	

Age at diagnosis (mean ± std dev)	49.8 ± 16.4	52.8 ± 15.4	47.7 ± 16.8	**0.006**
- Neuroendocrine/Carcinoid	40.1 ± 16.4	45.0 ± 15.5	37.3 ± 16.3	**0.01**
- Adenocarcinoma	55.8 ± 13.4	56.6 ± 14.0	55.1 ± 12.8	0.43
- Other	54.8 ± 12.3	51	55.3 ± 13.0	0.77

**Table 2:** Summary of incidence and histology of appendiceal cancers from Surveillance, Epidemiology and End Results (version 6.6.1)

	ALL CASES n = 3095	MALES n = 1489	FEMALES n = 1606
Incidence, per 100,000/year	0.4	0.4	0.4

Histology, n (%)			
- Neuroendocrine/Carcinoid	1002 (32)	408 (27)	594 (37)
- Adenocarcinoma	2062 (66)	1064 (71)	998 (62)
- Other	31 (1)	17 (1)	14 (1)

## Results and Conclusions

Very few case reports can be found in the literature regarding familial primary adenocarcinomas of the appendix. To our knowledge, the siblings reported here represent the first report of first degree relatives with appendiceal adenocarcinomas and the third report of familial appendiceal tumors [6,7]. The finding of polycystic kidneys in the two affected relatives is intriguing, but might be incidental.

Our efforts to find even a single additional familial case of appendiceal cancer among 314 Mayo cases were unsuccessful. While this was based only on a retrospective chart review, it suggests familial aggregation of appendiceal cancer is uncommon if not truly rare. We did document in the Mayo experience that adenocarcinomas are more common than neuroendocrine/carcinoid tumors of the appendix, in both men and women. This is in agreement with the SEER data. We also observed that the ages at diagnosis were older for the adenocarcinomas, and, for all appendiceal carcinomas, women were diagnosed on average about 5 years younger than men. We acknowledge the occurrence of these rare tumors within the siblings could be a result of chance alone but given the rarity of these tumors in the general population, the issue of familial predisposition seems still to remain a possibility. We were not able to systematically assess for cosegregation with any other type of cancer, given the nature of the chart review. Further investigation is warranted to explore potential hereditary predisposition.

## Consent

Written informed consent for use of the medical record was obtained from the patients for publication of this case report. A copy of the IRB documentation of authorization for use of the medical records for these two individuals is available for review by the Editor-in-Chief of this journal.

## Competing interests

The authors declare that they have no competing interests.

## Authors' contributions

AR did the primary literature research and drafted the manuscript. AP and MW provided clinical consultations, encouraged writing this report and provided final editing. NML consulted on the writing of the manuscript, audited some charts, reviewed the content, and edited the final product. KR performed statistical analysis and extracted the SEER data. All authors read and approved the final manuscript.
